# Nanogold-Lateral Flow Assay for Ginseng DNA Differentiation

**DOI:** 10.3390/bios15110757

**Published:** 2025-11-13

**Authors:** Al-Hashim Tiffere, Parsa Sojoudi, Christopher Oberc, Paul C. H. Li

**Affiliations:** Department of Chemistry, Simon Fraser University, Burnaby, BC V5A 1S6, Canada; al-hashim_tiffere@sfu.ca (A.-H.T.); parsa_sojoudi@sfu.ca (P.S.); mniico6@gmail.com (C.O.)

**Keywords:** gold nanoparticles, nitrocellulose membrane, single nucleotide polymorphism, DNA hybridization, spot test, lateral flow test

## Abstract

Different ginseng species, such as *Panax ginseng* and *Panax quinquefolius*, have various medicinal and economic values. Industrially and commercially, it is important to differentiate them. We have adopted the DNA hybridization method based on a single-nucleotide polymorphism (SNP) found in the ginseng DNA. The ginseng DNA samples were placed on a nitrocellulose membrane, and hybridization of the target sample with the probes immobilized on the membrane occurred, resulting in red spots for unaided eye visualization. We managed to demonstrate a spot test and then a lateral flow assay. Genomic DNAs were extracted from ginseng root samples and DNA amplification was used to generate the PCR products that flank the SNP site. We conclude that ginseng DNA can be differentiated based on a DNA lateral flow assay, detecting ~3 ng (in 1 μL) of PCR amplicons.

## 1. Introduction

Ginseng is a medicinal herb used for centuries in traditional medicine, particularly in Asia and North America [1,2]. Ginseng refers to the root of plants in the genus Panax, with the major types being Asian ginseng (*Panax ginseng*), native to Korea and China, and American ginseng (*Panax quinquefolius*), native to Canada and the US [3,4]. Asian ginseng (*Panax ginseng*) is highly valued for its adaptogenic properties, which help the body resist stress, maintain general body balance, improve mental health, and enhance sexual functions [3,5,6,7,8]. American ginseng (*Panax quinquefolius*) is also known for some other health benefits, such as boosting the immune system, reducing the risk of cancer, lowering blood sugar levels, especially in persons with type 2 diabetes, and inhibiting the growth of tumours [2,9,10,11].

Ginseng is sometimes called an adaptogen due to its ability to help the body deal with stress [5,6]. It contains ginsenosides, which are thought to give ginseng its medicinal properties [12,13,14,15,16]. Ginseng and its products are available in different forms: dried root, fresh root, standardized extract, tinctures, fluid extracts, powders, capsules, and tablets [17,18]. The two ginseng species (*Panax ginseng* and *Panax quinquefolius*) appear similar in many respects, though their therapeutic qualities and commercial values differ [7,19]. The similarities between the two ginseng species make their differentiation difficult, especially in their granular or powdered forms. Again, the similarities between the ginseng species make it unreliable to differentiate American ginseng and Asian ginseng using traditional means such as morphology, colour, texture, and other physical properties measurements [20,21]. This calls for the need to establish an adequate and simple procedure for distinguishing Asian ginseng from American ginseng.

Chemical methods are commonly used to differentiate between *Panax ginseng* (*P. ginseng*) and *Panax quinquefolius* (*P. quinquefolius*) [16,22,23]. Techniques such as liquid chromatography-mass spectrometry and capillary electrophoresis with UV detection are employed to measure the ginsenosides in the plant samples [15,16,21,23,24,25]. The Rg1/Re ratio can distinguish between the two species, with a high ratio indicating *P. ginseng* and a low ratio indicating *P. quinquefolius* [26,27,28]. Additionally, the type of polysaccharides in the sample can serve as a marker: *P. ginseng* contains more acidic polysaccharides like uronic acid, whereas *P. quinquefolius* has more neutral polysaccharides [29,30,31]. However, the limitations of these chemical methods include variability due to the need for a large amount of sample, as well as to the plant’s age and the part of the root sampled, all of which can influence the ginsenoside contents. Moreover, the freshness of the sample and storage conditions can also affect the results of these analyses [32]. 

On the other hand, the advances in genetic detection techniques have improved the identification and differentiation of biological species [12,18,33,34]. Genetic-based technique results are more reliable and have a high level of specificity, making the identification of closely related species like *P. ginseng* and *P. quinquefolius* much easier [35,36,37,38,39]. Several gene-based authentication techniques are available for ginseng differentiation [40,41,42,43,44,45], and they are based on polymerase chain reactions (PCRs), real-time PCRs, multiplex PCRs, or the high-resolution melting method, which demand the use of costly instruments. However, our focus is on the molecular method based on the low-cost method, i.e., the lateral flow test strip. The detection is based on single-nucleotide polymorphisms (SNPs) in the dammarenediol-II synthase (DS) gene of ginseng, which has been successfully used to differentiate ginseng species using PCR [26]. The differentiation of SNPs can be achieved using several methods, such as intron-targeting SNPs in the internal transcribed spacer (ITS) region, which is commonly used to identify ginseng species. However, gel electrophoresis is needed to analyze the products of PCR [46,47,48,49].

In this study, we have adopted the DNA-based lateral flow assay (LFA), as described in various reports [17,50,51,52,53], and we have designed a DNA probe to authenticate and differentiate between the DNA sequences of *P. ginseng* and *P. quinquefolius*. The LFA presented here is considered an effective method because it detects the genetic material (DNA), which is proven to be an efficient sample for biological species detection. The assay was performed on a nitrocellulose test strip by immobilizing a biotinylated capture probe via streptavidin-biotin binding, ensuring strong and stable probe attachment. A gold nanoparticle (AuNP)-labelled detection probe was used to generate a visible red signal upon successful hybridization with the target DNA, allowing results to be observed directly with the naked eye. The DNA-LFA is a rapid and simple diagnostic method that transports the target analyte to hybridize with the capture probe on the membrane via capillary flow. Compared to conventional molecular detection methods, the DNA-LFA is more accessible, faster, and user-friendly, requiring no specialized equipment or highly trained personnel. An additional advantage of LFA is that the presence or absence of a target analyte, such as DNA in the sample, is easily confirmed, since the test results are visible to the naked eye and require no need for a visualizing tool.

## 2. Experimental Section

### 2.1. Materials and Methods

All the DNA oligonucleotide sequences used for this study were obtained from Integrated DNA Technology (IDT) (Toronto, ON, Canada). Pierce^Tm^ Tris (2-carboxyethyl) phosphine hydrochloride (TCEP-HCl) was purchased from Thermo Fisher Scientific Co. (Ottawa, ON, Canada). Gold nanoparticles (AuNPs, 20 nm) were obtained from Sigma-Aldrich Corp (Oakville, ON, Canada). Streptavidin was obtained from Rockland Immunochemicals (Pottstown, PA, USA). Bundles of white matte backing cards used in the study were purchased from DCN Dx (Denoration) (Arlsbad, CA, USA). FF170HP nitrocellulose (NC) membrane (20 mm × 50 mm), sample and absorbent pads were purchased from Whatman (Maidstone, UK). As shown in the list of DNA oligonucleotides from Table 1, *P. ginseng* contains an A-T base pair at the SNP site, while *P. quinquefolius* contains a G-C base pair at the same position. These oligonucleotides were designed based on the SNP site, as reported previously [12,54].

### 2.2. Extraction of Genomic Ginseng DNAs and Their Amplification by PCR

A PCR product (126 bp) that encompasses the SNP site was generated. This was produced using P8′ and P7 primers (see Table 1), which were designed using the software DINAMelt 2005, as previously reported [54].

Two ginseng root samples were received, which are Chinese ginseng (ChG) and American ginseng (AmG). They were processed as previously reported [54]. Briefly, the samples were crushed into a fine powder, and the genomic DNA was extracted from these powdery samples (ca. 90 mg) using a Qiagen plant DNA extraction kit (Redwood City, CA, USA).

A PCR reagent kit (ABM, Richmond, BC, Canada) was used for the DNA amplification of the extracted DNAs. The PCR mixture (total volume 50 µL) comprised the following: 5 µL of 10X PCR buffer, forward and reverse primers (each of 10 µM, 1.5 µL), 10 µL of genomic DNA, MgSO_4_ (25 mM, 1.0 µL), dNTPs (10 mM, 1.0 µL), *Taq* polymerase (5 U/µL, 1.0 µL), and deionized (DI) water (29 µL).

The thermocycling was conducted in a thermocycler (Techne ^3^Prime, Canadawide Scientific, Ottawa, ON, Canada) using the following programme: (1) 3 min 94 °C initial denaturation, (2) 30 cycles of 30 s 95 °C denaturation, 30 s primer annealing (50 °C) and 30 s 72 °C elongation, and (3) final elongation at 72 °C for 3 min.

The PCR mixtures, which were subsequently purified using a Qiagen PCR purification kit, were quantified using UV spectroscopy (Nanodrop 2000, Mettler Toledo, Mississauga, ON, Canada), as previously described [54].

### 2.3. Design of DNA-LFA Test Strip

The LFA test strip includes the backing card, nitrocellulose membrane, sample pad, conjugate pad, and absorbent pad. First, the nitrocellulose membrane, which is attached to the backing card, provides the medium for the liquid flow and hybridization reaction. The conjugate and absorbent pads are then attached to the backing card at the two ends of the nitrocellulose membrane, creating an overlapping area on the nitrocellulose membrane to ensure continuous flow from one section of the test strip to another. The sample pad is also attached to the backing card, ensuring a continuous overlap on the conjugate pad. Each test strip is designed to measure 4 mm by 65 mm. In the study, another test strip, which is designed for a spot test, consists only of a backing card, nitrocellulose membrane, and an absorbent pad at each end.

### 2.4. Immobilization of Capture Probe

The capture probe, which is first immobilized on the membrane, is to hybridize with the target sequence. We used streptavidin-biotin’s strong binding affinity to prepare the biotinylated capture probe for immobilization on the nitrocellulose membrane. The immobilization solution comprised an equal amount (10 μL) of the DNA capture probe (Gin-bio or Quin-bio, 10 μM) and 1.25 mg/mL of streptavidin; this mixture was allowed to stand for 10 min to ensure efficient and uninterrupted interaction between the streptavidin and the biotin on the DNA capture probe. Then, 10 μL of 1X PBS buffer (137 mM NaCl, 2.7 mM KCl, 8 mM NaHPO_4_, and 2 mM KH_2_PO_4_) and 0.8 M CaCl_2_ were added to the mixture of capture probe and streptavidin. The solution (1 μL) was pipetted on the membrane for the immobilization of the streptavidin-bound captured probe.

### 2.5. Preparation of DNA–AuNP Conjugate

**Scheme 1:**
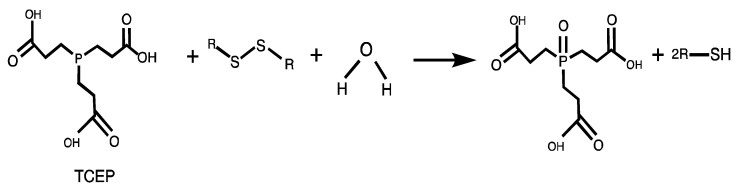
Reduction in oligonucleotide disulfide bonds (-SS-) to thiol (-SH) with TCEP.

DNA–AuNP conjugate preparation is the attachment of gold nanoparticles (AuNP) to the detection probe with the initial preparation of the thiol group (S-H) on the probe. This is performed by reducing the disulfide (S-S) bond on the detection probe to S-H using TCEP as a reducing agent. A TCEP-HCl (10 mM) solution was prepared by dissolving 0.887 g of solid TCEP in 9 mL of deionized water. The pH of the solution was determined to be around 2.5. The pH was adjusted to 7 by adding 3–4 drops of 10 M NaOH. A total volume of 10 mL was attained by adding deionized water. Before use, 1 mM of TCEP solution was prepared from a 10 mM TCEP solution by pipetting 1 mL of 10 mM TCEP solution into 9 mL of distilled water. The disulfide (S-S) was reduced to the thiol group (S-H) by mixing an equal amount of the detection probe (10 μM) and the TCEP (1 mM), and the mixture was allowed to stand at room temperature for 24 h. For AuNP attachment, 1 mL of 20 nm AuNP was added to the mixture and allowed to stand for 10–15 min; the mixture was centrifuged at 10,000 rpm for 10 min to give the pellet of AuNP-DNA conjugate. One mL of supernatant was carefully removed, the remaining pellet was resuspended in 1 mL of distilled water, and the centrifugation was repeated. The pellet of the second centrifugation (DNA–AuNP conjugate) was used for the experiments. The reaction between the TCEP and DNA with an S-S bond is shown in Scheme 1 below.

### 2.6. Image Analysis and Signal Quantification

After the hybridization experiments, the resulting signals were quantified based on their intensity. To accomplish this, ImageJ (version 1.54), a freely available and widely used image analysis software from NIH, was employed. ImageJ was used to process images of the test strips, captured with a smartphone camera in JPG or PNG formats. After transferring the images to the software, a region of interest (ROI) was selected around each assay spot using tools such as circles. The signal intensity within each ROI was then measured using ImageJ’s analysis functions, and the process was repeated for all assay spots.

The collected intensity data were organized and transferred to a spreadsheet software (e.g., Microsoft Excel) for further analysis. Statistical calculations, including mean values and standard deviations, were performed to compare spot intensities and assess hybridization efficiency. Data visualization through graphs and charts aided in interpreting the results. To ensure reliable and meaningful comparisons, all image processing and data analysis steps were carried out consistently across experiments.

## 3. Results and Discussion

### 3.1. DNA-Lateral Flow Assay Design

**Figure 1:**
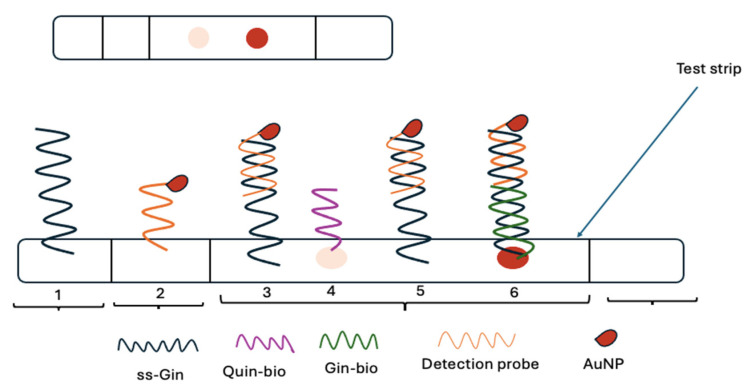
DNA lateral flow assay used to differentiate *P. ginseng* DNA from *P. quinquefolius* DNA. The numbers indicate the process occurring at each stage as the flow continues from left to right; for details, see text.

Figure 1 demonstrates how DNA-LFA is used to identify and differentiate *P. ginseng* from *P. quinquefolius* on a nitrocellulose membrane. The capture probe for *P. quinquefolius* (Quin-bio) is immobilized on spot 4, while the capture probe for *P. ginseng* (Gin-bio) is immobilized on spot 6. The target sequence (ss-Gin) on the sample pad at stage 1 flows to the conjugate pad at stage 2, where it is mixed with the DNA–AuNP conjugate, and the detection probe can hybridize with a part of the target sequence that is complementary to it. The solution continues to move along the membrane via capillary action to stage 3. The first spot at stage 4 with the capture probe, Quin-bio, is non-complementary to the remaining part of the target sequence, making it unable to hybridize with the target. As a result, no definite red colouration appears on spot 4. As the flow continues to the second spot at stage 6 with the capture probe Gin-bio, which is complementary to the remaining part of the target sequence that is not hybridized by the detection probe, it is captured and hybridizes with the target. Therefore, a clear red spot will be visible at stage 6 due to the AuNP-conjugate being retained even upon washing.

### 3.2. Determination of Successful DNA–AuNP Conjugate Formation

**Figure 2:**
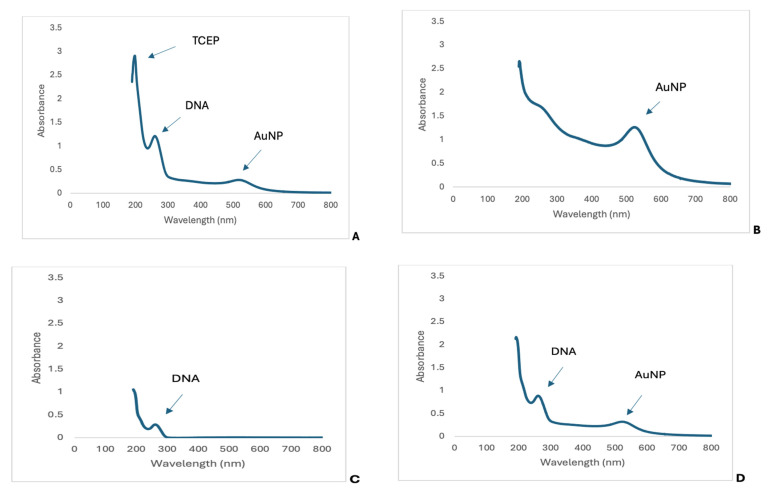
UV–Vis spectra for verification of the formation of the DNA–AuNP conjugate. (**A**) DNA–AuNP conjugate, (**B**) AuNPs only, (**C**) DNA only, (**D**) DNA/AuNPs mixture.

Proper attachment of the gold nanoparticles to the detection DNA probe is essential for the DNA-lateral flow assay detection; therefore, it is important to ensure that a strong and stable DNA–AuNP conjugate is successfully formed. There are various ways to determine a successful conjugate formation [50,51,52,53]. To this end, we used UV spectra analysis, ion-mediated aggregation of gold nanoparticles, and, finally, DNA hybridization on the membrane. DNA–AuNP conjugate, AuNP only, DNA only, TCEP only, and DNA/AuNP mixture were analyzed using UV–Vis. The UV–Vis spectral analysis results are shown in Figure 2A–D. The generated UV absorption peaks were analyzed to determine the components of the solution. From Figure 2A, the DNA–AuNP conjugate produced peaks at ~260 nm and 520 nm. These peaks are confirmed in Figure 2C,B to be DNA and AuNP, respectively, which are the components of the DNA–AuNP conjugate, after analyzing them separately. The presence of peaks representing DNA and AuNP in Figure 2A may be used to confirm the formation of the conjugate. However, we need further tests in order to confirm the successful binding of the AuNP to the detection DNA, and hence the successful formation of the conjugate.

To distinguish true conjugates from simple mixtures that display both DNA (260 nm) and AuNP (520 nm) absorbance peaks (see Figure 2D), a salt-induced aggregation assay was performed. In this method, sodium chloride (NaCl) was added to the DNA–AuNP conjugate, AuNP only, and DNA/AuNP mixture. The salt screens the electrostatic repulsion between nanoparticles, promoting their interaction and aggregation. This aggregation shifts the surface plasmon resonance peak toward longer wavelengths, producing a visible colour change from red to purple or blue [55].

This technique is used to confirm the successful binding of DNA to the AuNP surface. To perform the test, NaCl solutions of varying concentrations (50–200 mM) were prepared in deionized water and distributed into several vials. Each vial contained 450 μL of AuNP colloid, to which 50 μL of the NaCl solution was added, and the resulting colour changes were observed. The same DNA–AuNP conjugate used in Figure 2A was tested, and the outcomes are summarized in Table 2, highlighting the differences between the behaviours of bare AuNPs, AuNP–DNA mixtures, and true AuNP–DNA conjugates upon salt addition.

At NaCl concentrations above 100 mM, the gold nanoparticles (AuNP only) underwent aggregation. A similar effect was seen with the DNA–AuNP mixture, where the lack of DNA binding meant that only citrate ions contributed to AuNP stable dispersion; once the repulsive negative charges were screened by the added salt, the particles clumped together. In contrast, the DNA–AuNP conjugates behaved differently. The DNA strands attached to the nanoparticle surface contributed additional negative phosphate charges, which, along with citrate, stabilized the particles and prevented salt-induced aggregation by maintaining the electrostatic repulsion despite the compression of the electric double layer [56]. Consequently, no aggregation was observed in the DNA–AuNP conjugates.

**Table 2:** Tabulated results of the ion-mediated aggregation experiment shown in Figure 3. Salt concentration above 100 mM resulted in aggregation of the AuNP, causing a change in colour in both AuNP only and the DNA/AuNP mixture from red to pale blue. These results demonstrate the significance of DNA functionalization, or DNA–AuNP conjugate formation, in preventing AuNP aggregation in the presence of NaCl.

Solution	NaCl Concentration and Colour of Colloids					
	50 mM	80 mM	100 mM	130 mM	160 mM	200 mM
AuNP only	Red	Red	Red	Pale blue	Pale blue	Pale blue
DNA–AuNP conjugate	Red	Red	Red	Red	Red	Red
DNA/AuNP mixture	Red	Red	Pale blue	Pale blue	Pale blue	Pale blue

This process is often described as the “DNA functionalization” of gold nanoparticles. In essence, freshly synthesized gold nanoparticles exhibit high surface energy, which makes them prone to aggregation if only stabilized by citrate ions [57]. The attachment of DNA strands to their surface enhances stability by creating an electrostatic barrier that prevents nanoparticles from approaching each other too closely. When salts such as NaCl are added, the resulting increase in ionic strength reduces the repulsion provided by citrate [55]. However, the DNA coating supplies additional negative charges, maintaining repulsive forces between particles and thereby preventing their aggregation.

**Figure 4:**
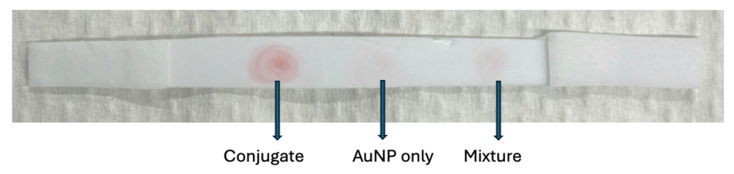
Results of DNA–AuNP conjugate, AuNP only, and DNA/AuNP mixture applied on a membrane. All three spots had bio-control immobilized, and only the spot with the conjugate remains red after washing with 1X PBS buffer.

The formation of the DNA–AuNP conjugate was also confirmed on the membrane using the principle of hybridization of two complementary DNA sequences. Here, the hybridization between the bio-control probe and the detection probe, which is attached to the AuNP, was used to confirm a stable and well-formed conjugate. The bio-control probe is complementary to the detection probe with the AuNP, and therefore their hybridization results in a red colour spot on the membrane after washing. The bio-control probe solution was prepared in the same way as the capture probe solution. The bio-control probe solution (1 μL) was applied to three different spots on the same membrane. Additionally, 1 μL of the DNA–AuNP conjugate, AuNPs only, and the DNA/AuNP mixture were added to three separate spots immobilized with the bio-control, as shown in Figure 4. The test strip was incubated at room temperature for 60 min before being washed with 1X PBS buffer. It was observed that only the spot applied with the conjugate remained red, while the AuNPs applied on the other two spots were washed away. The red spot at the location labelled “conjugate” appeared due to the hybridization between the bio-control probe and the detection probe, with the AuNP binding to it, i.e., the DNA–AuNP conjugate (see the left spot on Figure 4). The observation indicates a successful, stable conjugate formation. However, in the AuNP mixture containing the detection probe, though its hybridization with the bio-control probe could occur, AuNPs were washed off because they were not attached to the detection probe.

### 3.3. Spot Test Differentiation of P. Gin and P. Quin DNA

**Figure 5:**
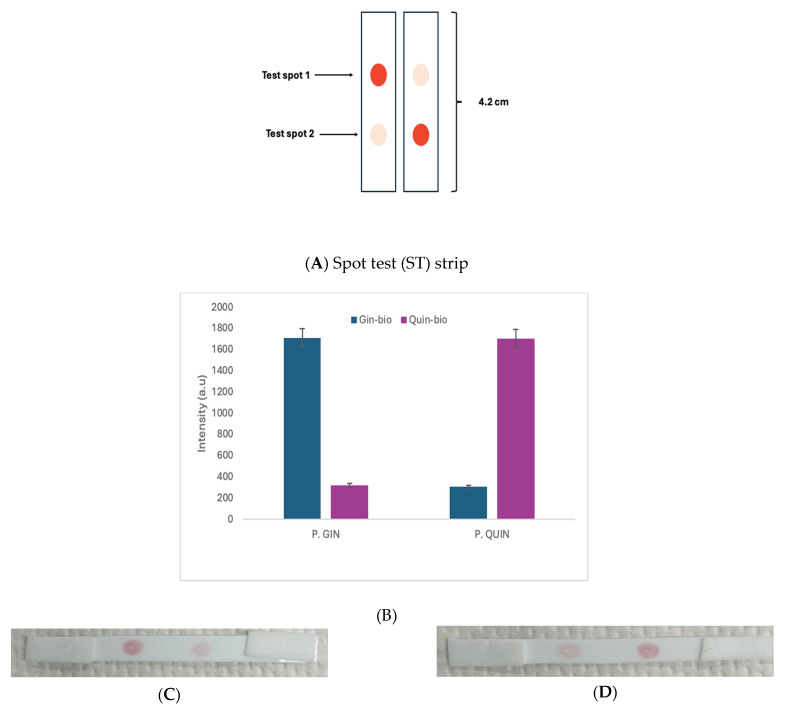
Spot test used to distinguish *P. ginseng* DNA sample from *P. quinquefolius* DNA sample. (**A**) Schematic diagram of the use of a test strip (4.2 cm) for the spot test. It was positive on test spot 1 for the left strip, but on test spot 2 for the right one. (**B**) Bar graph (n = 3) of differentiation of *P. gin* from *P. quin* DNA samples. (**C**) Image of *P. gin* DNA sample. (**D**) Image of *P. quin* DNA sample.

After the successful preparation and verification of a stable DNA–AuNP conjugate, we moved on to use it to conduct the spot test (ST) and DNA-lateral flow assay (DNA-LFA) for the detection and differentiation of the two similar ginseng DNA sequences from *P. ginseng* and *P. quinquefolius*. For the spot analysis, the sample solution was prepared by mixing equal amounts (10 μL) of the DNA–AuNP conjugate and the target sequence (10 μM), which is either ss-Gin or ss-Quin. The detection probe attached to the AuNP will hybridize to the portion of the target sequence it is complementary to, while the remaining portion of the target sequence will be hybridized by the appropriate immobilized probe on the membrane. This will generate a stable red colour signal that will be observed on the membrane as a red spot even after washing the membrane with 1X PBS solution (see Figure 5A). However, if the sample is applied to the wrong immobilized probe on the membrane, any red colour will disappear after PBS washing, leaving a faint pink-like spot, as seen in the images shown in Figure 5C,D. The signals from the images were quantified using an image analysis software (ImageJ) (see Figure 5B). This image analysis provides an in-depth interpretation of the signals generated.

From Figure 5B, the results indicate a successful differentiation of *P. gin* from *P. quin* using the spot test. The intensity values shown are averages of the replicates, and the error bars represent the standard deviations of three replicates. Figure 5C shows a spot test image for the *P. gin* DNA applied on the test strip. The spot on the left has a *P. gin* immobilized probe (Gin-bio), and the spot on the right has a *P. quin* immobilized probe (Quin-bio). A total of 1 μL of the prepared sample solution was pipetted onto each spot, and the test strip was incubated at room temperature for 30 min. The 30 min reaction time allowed efficient and stable hybridization to occur between the immobilized probe and the remaining portion of the target sequence not hybridized by the detection probe. In Figure 5C, the ss-Gin solution used on the spot with the *P. gin* immobilized probe (Gin-bio) has maintained a strong red colour signal even after washing with 1X PBS buffer. This occurs due to the hybridization of the target sequence (ss-Gin) and the appropriate immobilized probe (Gin-bio). The signal intensities, after washing the test strip with 1X PBS buffer and allowing it to dry for 10 min, were quantified using ImageJ software. From Figure 5D, the spot with the *P. quin* immobilized probe (Quin-bio) successfully hybridized with the *P. quin* target sequence (ss-Quin), producing a positive signal with a high signal intensity.

### 3.4. DNA-Lateral Flow Assay Detection of P. gin and P. quin DNA

**Figure 6:**
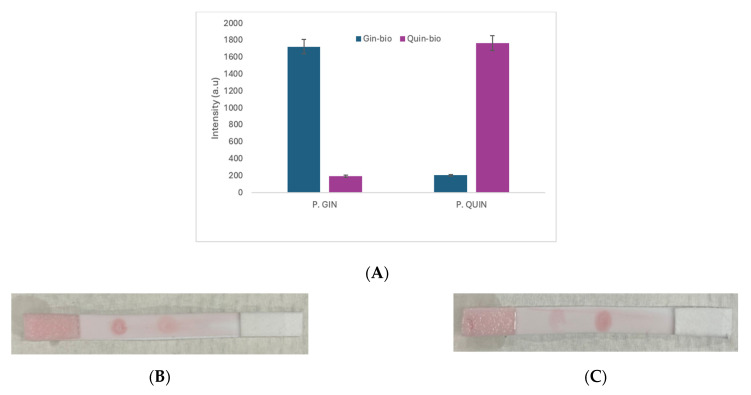
DNA-lateral flow assay without a conjugate pad used to distinguish *P. ginseng* DNA from *P. quinquefolius* DNA. (**A**) Bar graph (n = 3) for discrimination using ss-Gin and ss-Quin as the target DNAs. (**B**) Test strip image for the ss-Gin experiment. The diluted mixture of DNA–AuNP conjugate and target DNA is applied to the sample pad on the left. (**C**) Image for ss-Quin being the target DNA.

To conduct liquid flow in the lateral flow assay, one end of the test strip was placed in a test tube with a sample solution that contained either *P. gin* or *P. quin*’s target DNA (ss-Gin or ss-Quin) mixed with the DNA–AuNP conjugate. The sample solution for DNA-LFA analysis was prepared by mixing equal volumes (10 μL) of the conjugate with 10 μL of the ginseng target DNA (10 μM), either ss-Gin or ss-Quin. The mixture was allowed to stand for about 10 min, which allows the detection probe with the AuNP to hybridize with the portion of the target sequence it is complementary to. A total of 80 μL of nuclease-free water was added to bring the total volume to 100 μL. The left end of the test strip was placed in the solution, and the solution was allowed to flow upwards through the test strip due to capillary action. The remaining part of the target sequence, either ss-Gin or ss-Quin, was hybridized by the specific immobilized probe on the membrane on which the P. Gin-bio probe (left) and P. Quin-bio probe (right) were immobilized, as seen in the images provided in Figure 6. The flow was allowed to continue for up to 30 min. In our previous attempt, where the mixture of the DNA–AuNP conjugate and the sample solution was not diluted with 80 μL of water and was applied directly on the test strip, the entire test strip appeared red, and it was difficult to differentiate the spots from the background. However, the dilution of the sample and conjugate mixture here reduced the red colour intensity of the solution, and hence, the spots where DNA–AuNPs were captured were observed to have a red colour spot, creating a significant difference between the red spot and the background.

Figure 6 shows the results of the DNA-lateral flow assay. Figure 6A shows the signal intensities of the red spots analyzed after the images were taken with a smartphone camera. From Figure 6B, as the liquid sample flows, the target sequence (ss-Gin), together with the detection probe (DNA–AuNP), is captured at the spot containing the Gin-bio probe and produces an intense red colour spot (positive signal) upon washing. As the solution, which flowed up to the spot containing the Quin-bio probe, was not captured, a very faint red colour spot (negative) resulted. A similar result was obtained in Figure 6C, in which the sample solution contained ss-Quin, which is the target sequence for the Quin-bio probe. As the solution flows, the target sequence, together with the DNA–AuNP, gets to the Gin-bio spot before it gets to the Quin-bio spot. It is observed that the sample solution was not captured at the Gin-bio spot, leading to a very faint red colour spot being produced (negative). However, as the flow continued and arrived at the spot of the Quin-bio probe, which is the right capture probe, an intense red colour spot was produced (positive signal).

### 3.5. DNA-Lateral Flow Assay When the Target DNA Is Separated from the DNA–AuNP Conjugate

**Figure 7:**
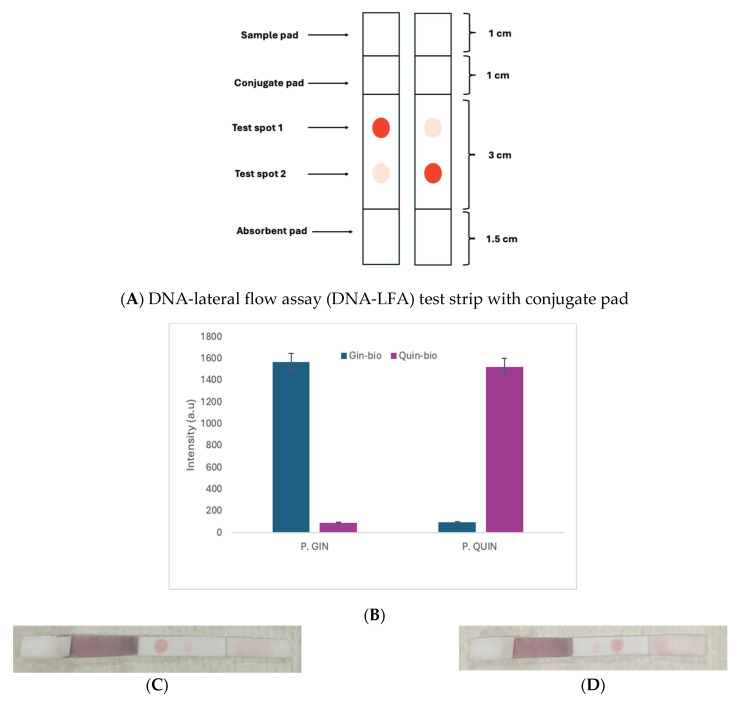
DNA-lateral flow assay with a conjugate pad (1 cm) used to distinguish the *P. ginseng* DNA from the *P. quinquefolius* DNA. (**A**) Schematic diagram of the use of a test strip (3 cm) for LFA. It was positive on test spot 1 on the left strip, but on test spot 2 on the right one. The DNA–AuNP conjugate has been prepared and dried on the conjugate pad. The diluted mixture of target DNA is applied to the sample pad (1 cm) to move toward the absorbent pad (1.5 cm). (**B**) Bar graph (n = 3) of the discrimination of ss-Gin and ss-Quin. (**C**) Test strip image of ss-Gin being the target DNA. (**D**) Image of ss-Quin being the target DNA.

In our attempt to improve the DNA-LFA detection and differentiation of *P. ginseng* and *P. quinquefolius* DNA with high specificity and precision, we separated the DNA–AuNP conjugate from the target sequence (ss-Gin). This was achieved by adding the conjugate pad to the test strip, and so the DNA–AuNP was not mixed with the target sequence, as shown in Figure 5 and Figure 6. Rather, the DNA–AuNP conjugate was placed on the conjugate pad, and it was allowed to completely dry up before attaching it to the backing card of the test strip. A total of 50 μL of the conjugate was used in making each conjugate pad. The sample solution was prepared by mixing 20 μL of the target sequence (ss-Gin or ss-Quin) and 80 μL of nuclease-free water, making a total volume of 100 μL sample solution. Figure 7 shows the results. The use of the conjugate pad significantly reduces the red background on the membrane and clearly distinguishes a positive sample from a negative sample compared to Figure 6. The sample pad of a test strip is completely immersed in the sample solution, and the sample solution is allowed to flow upwards due to capillary action. As the sample DNA reaches the conjugate pad, it picks up the conjugate by hybridizing with the detection DNA probe attached to the AuNP, and the flow continues along the membrane. The remaining portion of the target sequence is captured and hybridized by the appropriate immobilized probe, creating the red colour signal on the membrane. The sample, together with the conjugate, will not be captured on a spot with the wrong immobilized probe. The DNA-LFA detection is clearly demonstrated in Figure 7.

The signal intensities of the spots were analyzed using ImageJ, and the results are presented in the bar graph of Figure 7B. The signal intensity represents the red signals generated by the spots on the three membranes. In Figure 7, the positions of the immobilized probes for *P. ginseng* and *P. quinquefolius* in the test strip images are the same as those shown in the images in Figure 5 and Figure 6. In Figure 7C, the left end of the test strip is immersed in a test tube containing ss-Gin. As the sample flows to the conjugate pad, it picks up the DNA–AuNP conjugate and continues to flow. The sample, along with the conjugate, is captured at the first spot, which has the *P. ginseng* capture probe (Gin-bio), forming a distinct red spot (positive signal). However, the sample was not captured at the second Quin-bio spot. Thereafter, the unbound ss-Quin together with the conjugate was absorbed at the absorbent pad. The test strip was removed from the sample solution after 30 min. The opposite was clearly observed in Figure 7D, when the test strip was immersed in a test tube containing ss-Quin. The sample after picking up the conjugate could not be captured at the first Gin-bio spot. However, as samples continued to flow, it was captured at the second, which has the appropriate capture probe (Quin-bio), producing a red colour signal.

**Figure 8:**
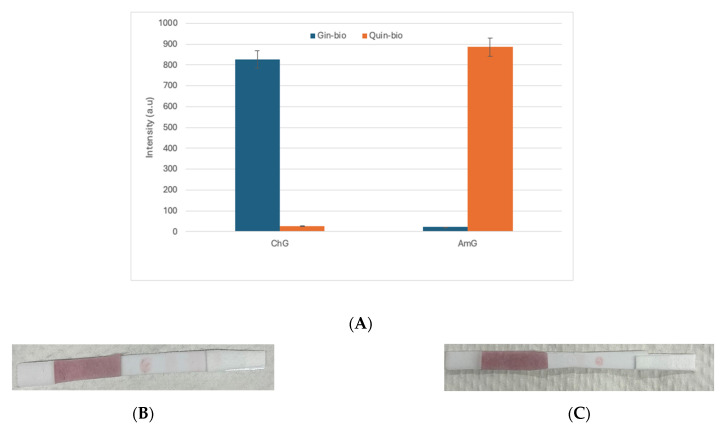
DNA-lateral flow assay with a conjugate pad used to distinguish the PCR product of *P. ginseng* (ChG) from that of *P. quinquefolius* (AmG). The DNA–AuNP conjugate has been prepared and dried on the conjugate pad. The target DNA is applied to the sample pad on the left. (**A**) Bar graph (n = 3) of the discrimination of ginseng PCR products. (**B**) Test strip image of ChG (2.8 ng) being the target DNA. (**C**) Image of AmG (3.2 ng) being the target DNA.

The method was then applied to DNA extracted from the root samples of *P. ginseng* and *P. quinquefolius.* The signal intensity graph in Figure 8A represents the red signals generated by the spots on three membranes, in which ChG binds mainly with Gin-bio and AmG binds with Quin-bio. The signal intensities of the spots (n =3) are presented in the bar graph. Figure 8B shows the test strip treated with the *P. ginseng* (ChG, 2.8 ng in 1 μL). As the sample flows to the conjugate pad, it picks up the DNA–AuNP conjugate and continues to flow. The sample, along with the conjugate, is captured at the first spot having the *P. ginseng* capture probe (Gin-bio), forming a distinct red spot (positive signal), but not at the second spot. Figure 8C shows the opposite case, when the *P. quinquefolius* sample (AmG, 3.2 ng in 1 μL) was used. From the test strip image, the sample after picking up the conjugate could not be captured at the first Gin-bio spot, but was captured at the second, which has the appropriate capture probe (Quin-bio), producing a red colour signal.

## 4. Conclusions

The developed DNA-lateral flow assay successfully identified and distinguished *P. ginseng* from *P. quinquefolius* DNA samples, which differ from each other by only one SNP. The method does not require any equipment, and, with the help of AuNPs, the results are visible to the naked eye within 30 min after perfectly matched hybridization. This was achieved after designing probes based on a reported SNP site with a single nucleotide difference in the ginseng DNA, and the implementation of the lateral flow assay. This method could be extended to genetically identify other biological species’ DNA with close similarities.

## Figures and Tables

**Figure 3:**
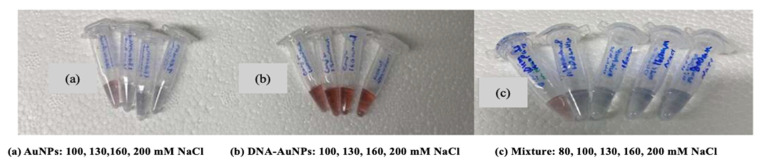
Ion-mediated aggregation experiment results: (**a**) AuNPs only: colour changes from red to blue after adding NaCl (130, 160, and 200 mM), indicating AuNPs aggregation. (**b**) DNA–AuNPs conjugate: no colour change observed with NaCl (100, 130, 160, and 200 mM), as DNA-functionalized gold nanoparticles prevent aggregation at concentrations ranging from 100 mM to 200 mM. (**c**) DNA–AuNP mixture: colour change observed after adding 100, 130, 160, and 200 mM NaCl, indicating aggregation occurs.

**Table 1:** DNA oligonucleotide sequences and their modifications for both *P. ginseng* and *P. quinquefolius*. The bold and underlined nucleotides indicate the base pairing at the SNP site.

Probe	Sequence (5’-3’)	Modification
Gin-bio (*P. ginseng* capture probe)	CTAAAAAAAAAGTATTT**T**TCATCTAAATTTT GAA	5’-biotin
Quin-bio (*P. quinquefolius* capture probe)	CTAAAAAAAAAGTATTT**C**TCATCTAAATTTT GAA	5’-biotin
ss-Gin (*P. ginseng* sample)	TGA AAA TCA ATT TAA GAC ACT TTC AAA TTC AAA ATT TAG ATG A**A**A AAT ACT TTT TTT TTA	5′-phosphate
ss-Quin (*P. quinquefolius* sample)	TGA AAA TCA ATT TAA AAC ACT TTC AAA TTC AAA ATT TAG ATG A**G**A AAT ACT TTT TTT TTA	5′-phosphate
Detection probe	TTA AAT TGA TTT TCA	3’ disulfide (S-S)
Bio-control	TGA AAA TCA ATT TAA	5’-biotin
P7 forward primer	ATGTTTGT TACTCCCTCC GTT	5′-phosphate
P8′ reverse primer	TACAGTGAT AATTAAATAT TGTAACTATC TAA	5′-phosphate

## Data Availability

The original contributions presented in this study are included in the article. Further enquiries can be directed to the corresponding author.
